# Aligned electrospun fiber film loaded with multi-enzyme mimetic iridium nanozymes for wound healing

**DOI:** 10.1186/s12951-022-01685-2

**Published:** 2022-11-16

**Authors:** Boda Wu, Jintao Yang, Yan Zu, Junjie Chi, Keqing Shi

**Affiliations:** 1 Translational Medicine Laboratory, The Center of Wound Healing and Regeneration, The First Affiliated Hospital of Wenzhou Medical University, Wenzhou, 325035 China; 2 Oujiang Laboratory (Zhejiang Lab for Regenerative Medicine, Vision and Brain Health), Wenzhou, 325001 Zhejiang China; 3 Engineering Research Center of Clinical Functional Materials and Diagnosis & Treatment Devices of Zhejiang Province, Wenzhou Institute, University of Chinese Academy of Sciences, Wenzhou, 325024 Zhejiang China; 4 Cixi Biomedical Research Institute, Wenzhou Medical University, Wenzhou, 325035 Zhejiang China; 5 Key Laboratory of Intelligent Critical Care and Life Support Research of Zhejiang Province, The First Affiliated Hospital of Wenzhou Medical University, Wenzhou, 325035 China

**Keywords:** Electrospun fiber film, Hydrogel, Iridium nanozymes, Silver nanoparticles, Wound healing

## Abstract

A film with elaborate microstructures that offers biomimetic properties and multi functionalities is highly desired in wound healing. Here, we develop an aligned hydrogel fiber film integrated with multi-active constituents to promote wound healing. Such fiber films are designed and constructed by photo-crosslinking the methacrylate gelatin (GelMA) doped with silver nanoparticles (Ag NPs) and iridium nanoparticles coated with polyvinylpyrrolidone (PVP-Ir NPs) in the precursor solution using electrospinning. The nature of GelMA hydrogel and the aligned arrangement of nanofibers endow the film with high-water content, self-degradability, improved bionic characteristics, oriented cell growth, and improved cell proliferation and migration. Moreover, the encapsulated nanozymes and Ag NPs offer the fiber film with superior reactive oxygen species (ROS) scavenging and antibacterial capability. The infected wound model shows that the multi-active hydrogel fiber film can reduce inflammation by killing bacteria and decomposing ROS, which accelerates the growth of new blood vessels and granulation tissue. Benefitting from these features, the versatile aligned GelMA fiber film demonstrates the clinically translational potential for wound healing.

**Supplementary Information:**

The online version contains supplementary material available at 10.1186/s12951-022-01685-2.

## Introduction

The skin, being the sizeable part of the human body, makes every effort to protect the body from the external stimuli. Its functions of physical insulation, immune protection, and metabolism will be severely impaired once the skin is wounded without its intact structure [1, 2]. Production in large quantities of ROS at the wound site [3, 4], have been demonstrated to be critical factors that suppress the wound healing. Although the body can initiate the physiological protection system with the synthesis of various antioxidant enzymes to moderate the increased level of ROS, the excessively ROS production may overwhelm the physiological antioxidant capacity and hinder the curing process [5, 6]. Moreover, continuous infection is one of the factors hindering wound healing. Traditionally, wound dressings, including gauzes and patches, have been widely adopted in clinical treatments [7–9]. However, limitations still exist in applications, such as rejection or allergic reaction, infection potential after soaking, comparatively single structure, and limited functionality [10]. Besides, bioactive reagents used for ROS scavenging, such as natural enzymes and antioxidant molecules, present fatal defects of low stability, complicated synthesis, and short half-life in vitro [11]. And the commonly used antibiotics trends to induce bacterial resistance. Therefore, a newly designed dressing with a multifunction and refined structure is urgently needed to overcome the aforementioned hurdles.

In this research, we proposed an aligned methacrylate gelatin (GelMA) electrospun fiber film with polyvinylpyrrolidone coated iridium nanozymes (PVP-Ir NPs) and silver nanoparticles (Ag NPs) load to promote wound curing. Electrospun fiber film has been widely used owing to their similarities with the extracellular matrix (ECM) in tissue regeneration engineering [12–17]. In addition, the high surface-volume ratio and porous nature of the ECM-like fiber film enable cell-material interactions and facilitate nutrient-waste exchange [18]. However, the current fiber film possesses neither water retaining capability nor the designed microstructure, hindering its further applications in wound curing. Recently, photocrosslinkable GelMA hydrogel, incorporating methacryloyl groups into gelatin, exhibits hydrophilicity and elastic compliance characteristics, making it an excellent candidate for bionic applications [19–22]. In addition, artificial enzymes, also known as nanozymes, have been recognized for their superior performance, such as high catalytic activity, facile production, high stability, and economic benefits, over natural enzymes and traditional antioxidant molecules [23–25]. Among them, iridium-based nanoparticles showed multi-enzymes mimic ability and excellent ROS scavenging efficiency with lower systemic toxicity, which have proved their values in biomedical detection, cancer therapy, and other fields [26, 27]. However, integrating such nanozymes in wound healing is still at the infant stage.

**Scheme 1.**
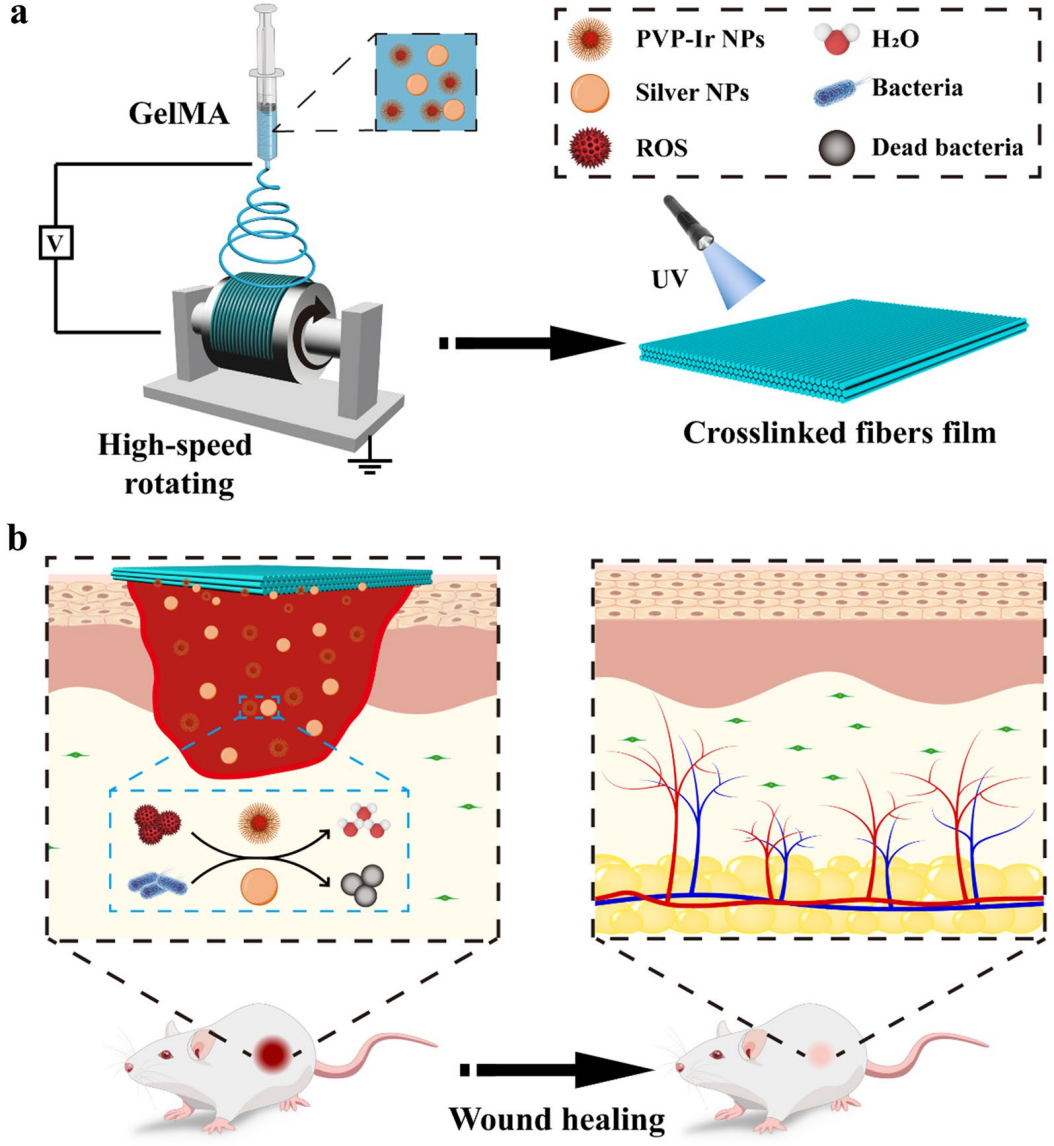
**a** Preparation process of the electrospinning aligned GelMA fiber film preparation process. **b** Bionic application of the aligned fiber film in wound healing.

Herein, we combined the PVP-Ir NPs and Ag NPs with biocompatible GelMA hydrogel to develop an aligned fiber film to promote wound healing, as presented in Scheme 1. The functional aligned fiber film was developed by electrospinning, where the precursor solution was collected via a high-speed rotating collector and photo-crosslinking afterwards. The obtained film composed of GelMA hydrogel showed a high-water content and biocompatibility, which improved cell adhesion and proliferation. Meanwhile, the ordered arrangement of the fibers in the film could induce the directional growth of cells [28–30]. Furthermore, by integrating PVP-Ir NPs into pre-gel as catalytic moieties, the prepared fiber film had a scavenging ability against various types of ROS. Finally, the encapsulated Ag NPs generated an antibacterial dressing for the fiber film [31, 32]. In vivo experiments that demonstrated the multifunctional aligned fiber film could prevent bacterial infection and accelerate the healing process were conducted, which removed ROS generated at the wound site and improved granulation tissue and new blood vessel growth. The aligned GelMA hydrogel fiber film showed great potential for wound curing due to the features mentioned above.

## Results

### Preparation and characterization of PVP-Ir NPs and composite fiber films

**Fig. 1:**
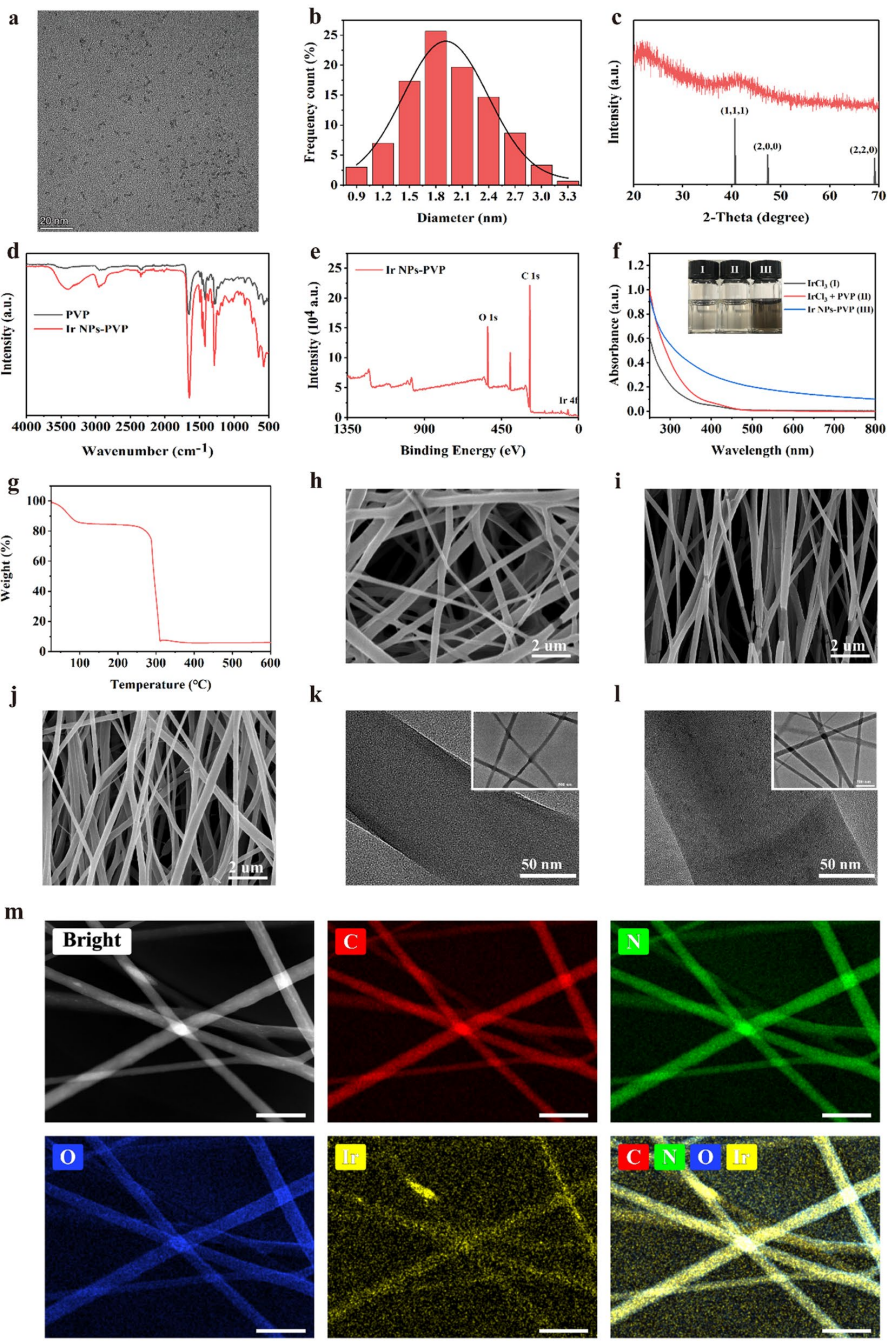
**a** TEM results of synthesized nanozymes. **b** Size distribution analysis of 300 random nanoparticles. **c** XRD results of nanozymes. **d** FTIR analysis of PVP and PVP coated nanozymes. **e** XPS results of nanozymes. **f** UV spectra analysis of various solutions. The insert shows the photos of the IrCl_3_ (I), IrCl_3_ + PVP (II), PVP-Ir NPs (III). **g** TG analysis of PVP-Ir NPs. SEM image of **h** GelMA/R, **i** GelMA/A, **j** Ir@GelMA/A. TEM images of **k** GelMA fibers, and **l** Ir@GelMA fibers. **m** EDS analysis of PVP-Ir NPs loaded GelMA fibers. The scale bars are 20 nm in **a**, 2 μm in **h**, **i**, and **j**, 50 nm in **k**, **l**, and **m**, and 500 nm in the insert images

In experiments, PVP-Ir NPs were synthetic based on the previous reports with minor modifications. Briefly, the precursor iridium trichloride hydrate was first dissolved in H_2_O and treated by ultrasound until a clarified solution was obtained. Then the solution was added into the PVP contained ethanol solvent and stirred overnight at room temperature. After that, a bright yellow solution was acquired and then refluxed for another 8 h at 100 ℃. The resulting brown solution was treated by rotary evaporation to remove the solvent, and black production was obtained at the end. Transmission electron microscopy (TEM) demonstrated that the PVP-Ir NPs showed a subspherical structure, and no apparent aggregation was observed (Fig. 1a). Meanwhile, the statistical analysis of 300 nanoparticles based on TEM images showed that most PVP-Ir NPs distributed at 1 to 3 nm diameter with an average size of approximately 1.8 nm (Fig. 1b). The diffraction peaks at 40.66°, 47.32°, and 69.14° were sharp and intense based on the results of X-ray diffraction (XRD) analysis, indicating the crystal-like structure of the particles (Fig. 1c), which is consistent with the JCPDS NO. 06–0598. The successful surface coating by PVP was confirmed by the Fourier transform infrared spectra (FT-IR) (Fig. 1d), which have a characteristic absorbance peak at 1286 and 1657 cm^−1^ for C–N and C=O bonds. Furthermore, X-ray photoelectron spectroscopy (XPS) identified the electronic state of Ir_(0)_, which contributed to the catalytic mechanism of iridium nanozymes. And successfully coordinated pyrrole groups of PVP to nanozymes was also confirmed based on the results of XPS (Fig. 1e). Furthermore, the UV–vis spectrum and the corresponding color changes of the solution verified the fabrication of nanozymes (Fig. 1f). Additionally, the Thermogravimetric analysis (TGA) further demonstrated the PVP exiting, with an almost 80% weight ratio in PVP-Ir NPs (Fig. 1g).

To obtain the hydrogel fiber film with desirable microstructure and multi-functions, photocrosslinkable GelMA was electrospun with a rotating collector with variable speeds. The obtained GelMA fibrous films with random or aligned fibers arrangement were named GelMA/R and GelMA/A, respectively, while the one with aligned fibers and loaded PVP-Ir NPs was named Ir@GelMA/A. Scan transmission electron microscope (SEM) indicated that the random and aligned crosslinked microstructure of hydrogel fiber films could be formed under different collector rotation speeds (Fig. 1h–j). Based on the SEM results, the diameters of random fibers were distributed between 250 and 450 nm (Additional file 1: Fig. S1a). The high collector rotating speed of about 2500 rpm/min could reduce the diameter of fibers in the aligned groups, similar to the previous literature (Additional file 1: Fig. S1b). However, the doping of PVP-Ir NPs into the aligned fibers under the high-speed condition increased the average diameter of fibers (Additional file 1: Fig. S1c). TEM and EDS analysis demonstrated the successful encapsulation of PVP-Ir NPs (Fig. 1k–m). The GelMA fibers showed a clean interior without nanoparticles loaded (Fig. 1k). The black dots, however, were distributed in the fibers after electrospinning (Fig. 1l). The EDS analysis showed the homogeneous distribution of Ir elements in the fibers (Fig. 1m), referring to the successful encapsulation of PVP-Ir NPs. Furthermore, the results of ICP-MS demonstrated the concentration-dependent loading ability of PVP-Ir NPs (Additional file 1: Fig. S2).

### *Electrospun fiber film loaded with iridium nanozymes simulates multiple antioxidant enzymes *in vitro

**Fig. 2:**
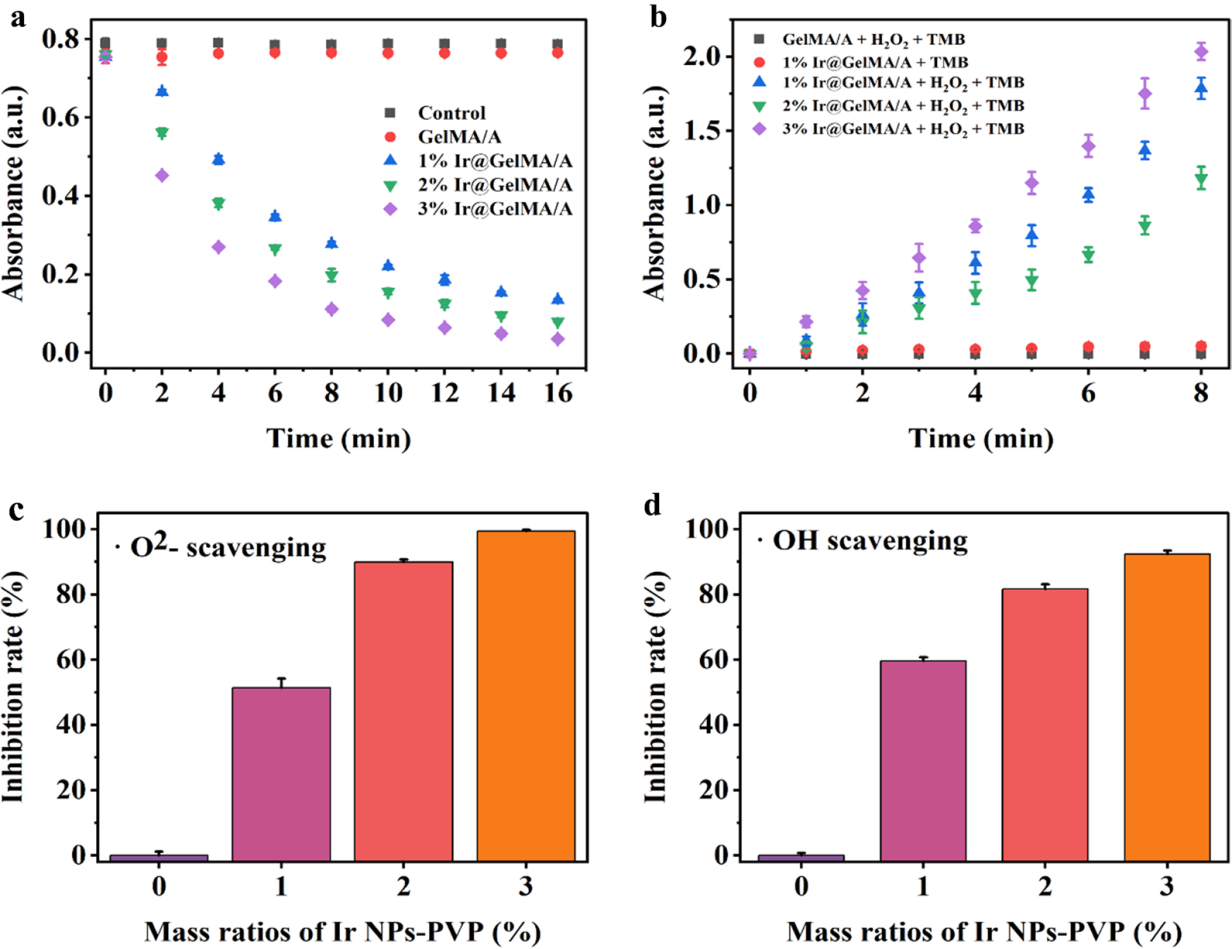
**a** CAT-like activity of various mass ratios of Ir@GelMA/A (n = 3 for each group). **b** POD-like activity of various ratios of Ir@GelMA/A (n = 3 for each group). **c** SOD-like activity of various mass ratios of Ir@GelMA/A (n = 3 for each group). **d** Hydroxyl radicals scavenging effect of various mass ratios of Ir@GelMA/A (n = 3 for each group)

To investigate the capability of ROS scavenging, a chains of enzyme activity experiments were applied to certify the multi-enzymes mimicking effect of various concentrations of PVP-Ir NPs loaded in the aligned GelMA hydrogel fiber films, namely 1%, 2%, and 3% Ir@GelMA/A, respectively. First, the CAT-like activity was tested by reducing H_2_O_2_ through the UV absorption spectrum at 240 nm. The results indicated a time-dependent and concentration-dependent H_2_O_2_ degradation by the Ir@GelMA/A groups, while no apparent changes were observed between the Control and pure GelMA/A groups, as shown in Fig. 2a. Next, the commercial reagent Catalase was set as a positive control, demonstrating the CAT-like activity of Ir@GelMA/A (Additional file 1: Fig. S3). Considering the production of oxygen from H_2_O_2_ under the catalysis of CAT, the generation of bubbles and the floating of films further demonstrated the CAT-like of Ir@GelMA. In contrast, pure GelMA/A film stayed at the bottom during the experiments (Additional file 1: Fig. S4). To directly confirm the production of oxygen under the catalysis of CAT-like activity, we performed a dissolved oxygen test to clarify the generated oxygen. The results showed that the concentration of the oxygen increased as the catalysis proceeded, while no apparent changes were observed in the control and the pure fiber film groups (Additional file 1: Fig. S5). To explore the POD mimicking activity of Ir@GelMA/A, the 3,3′,5,5′-tetramethylbenzidine (TMB) was utilized to evaluate the catalysis effect. The significant time-dependent and concentration-dependent changes of the absorption values of TMB-ox at 652 nm confirmed the POD-like of the fiber films encapsulated with PVP-Ir NPs. However, the absorption value of TMB-ox was nearly unchanged without H_2_O_2_ or PVP-Ir NPs loading, respectively (Fig. 2b).

Superoxide is also the common ROS generated in vivo. Thus, the superoxide dismutase kit was used to detect the SOD mimetic ability of fiber films containing different concentrations of the nanozymes. The Ir@GelMA/A fiber films showed a concentration-dependent relationship of superoxide radicals scavenging ability with approximately 51, 89, and 99% of the •O_2_^−^ decomposed by 1%, 2%, and 3% PVP-Ir NPs loaded within 60 min, respectively (Fig. 2c). Moreover, almost 92% of hydroxyl radical (•OH) was decomposed by 3% Ir@GelMA/A, which showed high sensitivity to •OH (Fig. 2d). These results indicated that Ir@GelMA/A possessed multi-enzymes mimetic ability against various ROS.

### Aligned electrospun fiber film loaded with iridium nanozymes eliminates intracellular ROS and induces cell orientation growth

**Fig. 3:**
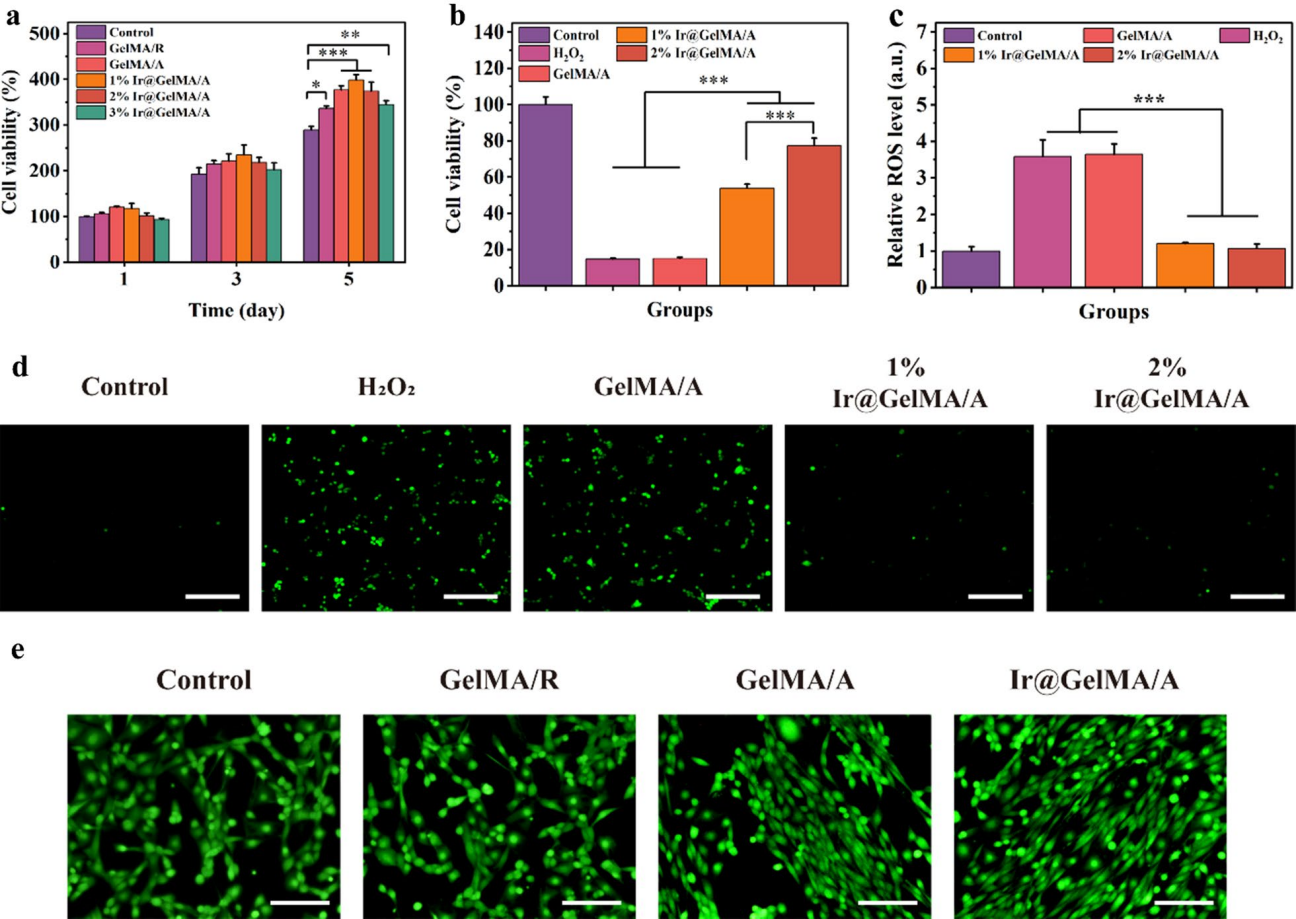
**a** The cell viability of NIH-3T3 after different treatments. **b** The cell protective effect of PVP-Ir NPs loaded fiber film against H_2_O_2_ treatment. **c** The flow cytometry quantification of cellular ROS stained by DCFH-DA. **d** Fluorescence photos of the intracellular ROS. **e** The effect of inducing cell orientation growth of various GelMA fiber films. The scale bars are 200 μm in **d**, and **e**

H_2_O_2_ and O^2−^ are the most plentiful ROS produced intracellular during the wound process. Physiologically, cells can generate multifarious antioxidant enzymes stimulated by moderate oxygen stress to scavenge ROS. However, the overwhelming production of ROS may break this harmony and lead to cell death. Encouraged by the aforementioned characteristics of Ir@GelMA/A, we evaluated their potential ability to scavenge ROS at the cellular level and cytoprotective effect*.* First, we tested the cytocompatibility of synthesized fiber films. As shown in Fig. 3a, NIH-3T3 cells on the GelMA hydrogel fiber film had better viability and proliferation than the cells on the culture plate. These differences were due to the good biocompatibility and better physiological environment provided by GelMA hydrogel for cell proliferation. Furthermore, the orientated structure of fiber film made the cell adhesion easier and grew faster, thus having an improved proliferation in the GelMA/A, 1% GelMA/A, and 2% GelMA/A groups than the GelMA/R group. However, fiber film loaded with 3% PVP-Ir NPs slightly impeded the viability of cells. Afterward, the antioxidant ability of various fiber films was analyzed. Contrasted with the Control or GelMA/A groups, the proliferative activity of the cells was promoted coculturing with 1% and 2% Ir@GelMA/A (Fig. 3b), suggesting the significant protective ability of Ir@GelMA/A against oxidative stress injury. In addition, the quantification of the cellular ROS by the flow cytometry experiments certified the excellent ROS scavenging activity of fiber film loading with PVP-Ir NPs (Fig. 3c). Furthermore, the fluorescence micrography indicated an elaborated ROS level after H_2_O_2_ treatment, in which prominent decreases were observed both in 1% and 2% Ir@GelMA/A treated groups (Fig. 3d). Then, we assessed the ability of aligned fiber film to induce cell orientation. The obtained fluorescence images revealed that cells seeded on GelMA/R grew disorderly. On the other hand, the directional growth of cells along the nanofibers was observed on GelMA/A fibrous film. Moreover, the ability of the GelMA/A fiber film to induce orientation was not impaired with 2% PVP-Ir NPs loaded (Fig. 3e). And the angle distribution analysis of cells further demonstrated the conclusions mentioned above (Additional file 1: Fig. S6). Therefore, the 2% Ir@GelMA/A fibrous film was chosen for further experiments based on the results mentioned above.

### Composite fiber film loaded with silver nanoparticles inhibits bacterial growth

**Fig. 4:**
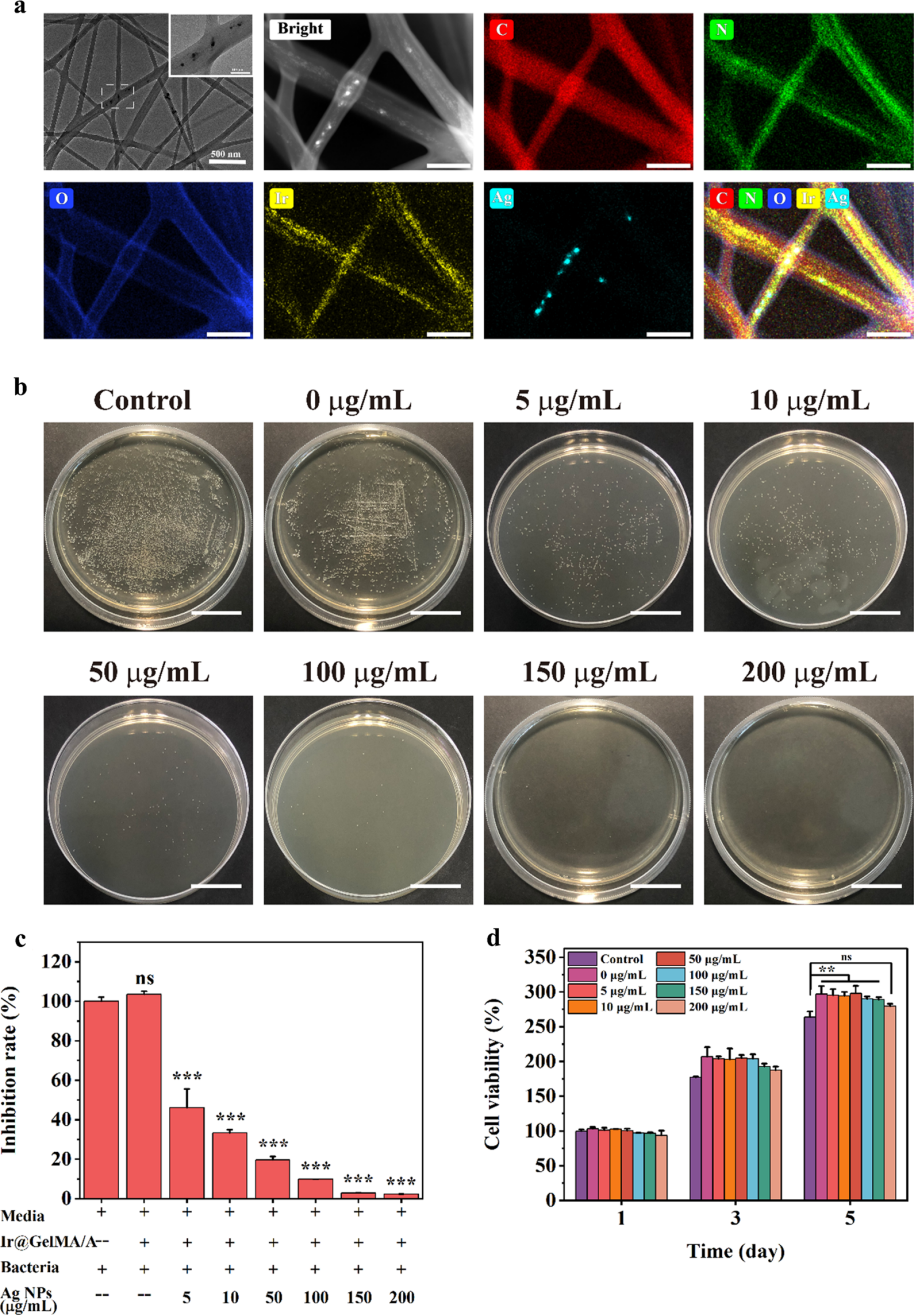
**a** TEM images and EDS analysis of Ir@Ag@GelMA fibers. **b** The antibacterial effect against *S.aureus* of various concentrations of Ag NPs loaded Ir@Ag@GelMA fibrous films. **c** Quantification analysis of antibacterial effect. **d** The biocompatibility of various concentrations of Ag NPs and 2% PVP-Ir NPs loaded GelMA fiber film. The scale bars are 200 nm in **a**, and 200 mm in **b**

Adding the various concentrations of Ag NPs to the pre-electrospinning solution, we obtained Ag NPs and PVP-Ir NPs co-loaded in the aligned GelMA fiber films, named as Ir@Ag@GelMA/A. The large nanoparticles and small dots observed in the TEM images and the analysis of the corresponding elements indicated the simultaneous loading of both nanoparticles (Fig. 4a). To clarify the potential antibacterial activity of the multifunction GelMA hydrogel fiber film, gram-positive *Staphylococcus aureus* (*S.aureus*) were selected to coculture with various concentrations of Ag NPs (0–200 μg/mL) loaded fiber films. The results of bacterial colony count and quantification analysis showed that the *S.aureus* in the PBS and Ir@GelMA/A groups maintained normal viability. Furthermore, the inhibition rate against the bacterial was positively associated with the loading concentrations of Ag NPs. Ir@GelMA/A fiber film with about 150 μg/mL Ag NPs loaded could inhibit the growth of *S.aureus* completely (Fig. 4b, c). In addition, we also demonstrated the biocompatibility of Ag NPs loaded GelMA fiber film. The cell-material coculture experiments showed good viability and proliferation of NIH-3T3 cells seeded on different fiber films similar to that seeded on the plastic plate, while with slightly compliance to the viability in the 200 μg/mL group (Fig. 4d). Moreover, the SEM images showed the addition of such nanoparticles did not affect the aligned structure of the fibers in the Ir@Ag@GelMA/A group (Additional file 1: Fig. S7), and the correspondingly cell experiments further demonstrated that the Ir@Ag@GelMA/A can still markedly induce the orientation growth of cells as Ir@GelMA/A did (Additional file 1: Fig. S8). Thus, we chosen the 2% PVP-Ir NPs and 150 μg/mL Ag NPs loaded Ir@Ag@GelMA/A for the further experiments.

### Multifunctional composite fiber film has admirable water retention ability, self-degradation property, and improved mechanical strength

**Fig. 5:**
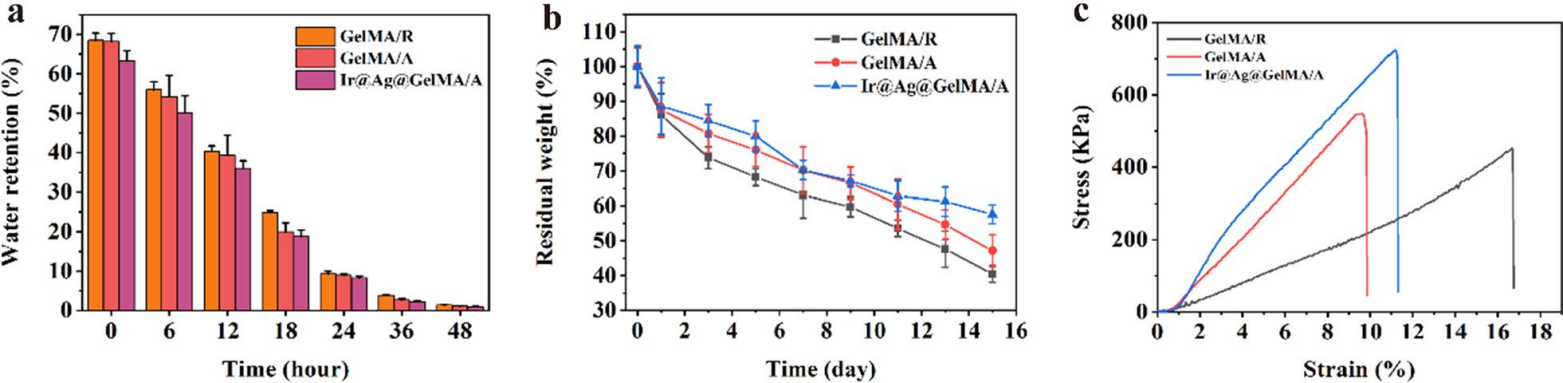
**a** Water retention properties of GelMA/A and Ir@Ag@GelMA/A (n = 3 for each group). **b** Degradation of hydrogel fiber film in vitro (n = 3 for each group). **c** The stress–strain curve of various hydrogel fiber films

A desirable dressing should improve the exchange of nutrients and metabolites with good water absorbance and retention ability for wound healing. Thus, we evaluated the wettability of the GelMA fiber film. As the results showed, the GelMA/R and GelMA/A fiber film could soak up water for almost 70 times its original weight, and the water was retained over 48 h. Moreover, the nanoparticles loaded did not impaired the swelling and water retention properties (Fig. 5a). The dressing used in wound healing provides temporary support and biological effects for cell adhesion and proliferation but degrades after activating the tissue growth. Thus, the degradation rate of the obtained fiber film was further investigated in vitro simulation experiments. Owing to the hydrophilicity nature of GelMA hydrogel, the fiber film degraded slowly (Fig. 5b). However, the Ir@Ag@GelMA/A group had the lowest degradation rate, while the GelMA/R degraded the fastest. The results demonstrated the biodegradability of the obtained GelMA fibrous films, which contributed to the biosafety used in vivo. After in vivo application of the hydrogel fiber film, the capacity to resist deformation is crucial for maintenance of structural stability. Therefore, the tensile test was applied to testify the mechanical strength of various fiber film. Results showed that all the GelMA fiber films exhibit the typical stress–strain curve of viscoelastic materials. Among them, GelMA/R had the lowest mechanical strength and the longest elongation at break. The aligned structure and the encapsulation of nanoparticles correspondingly increased the tensile strength of the fiber film (Fig. 5c).

### The aligned fiber films induce increased cell migration in a wound-healing test

**Fig. 6:**
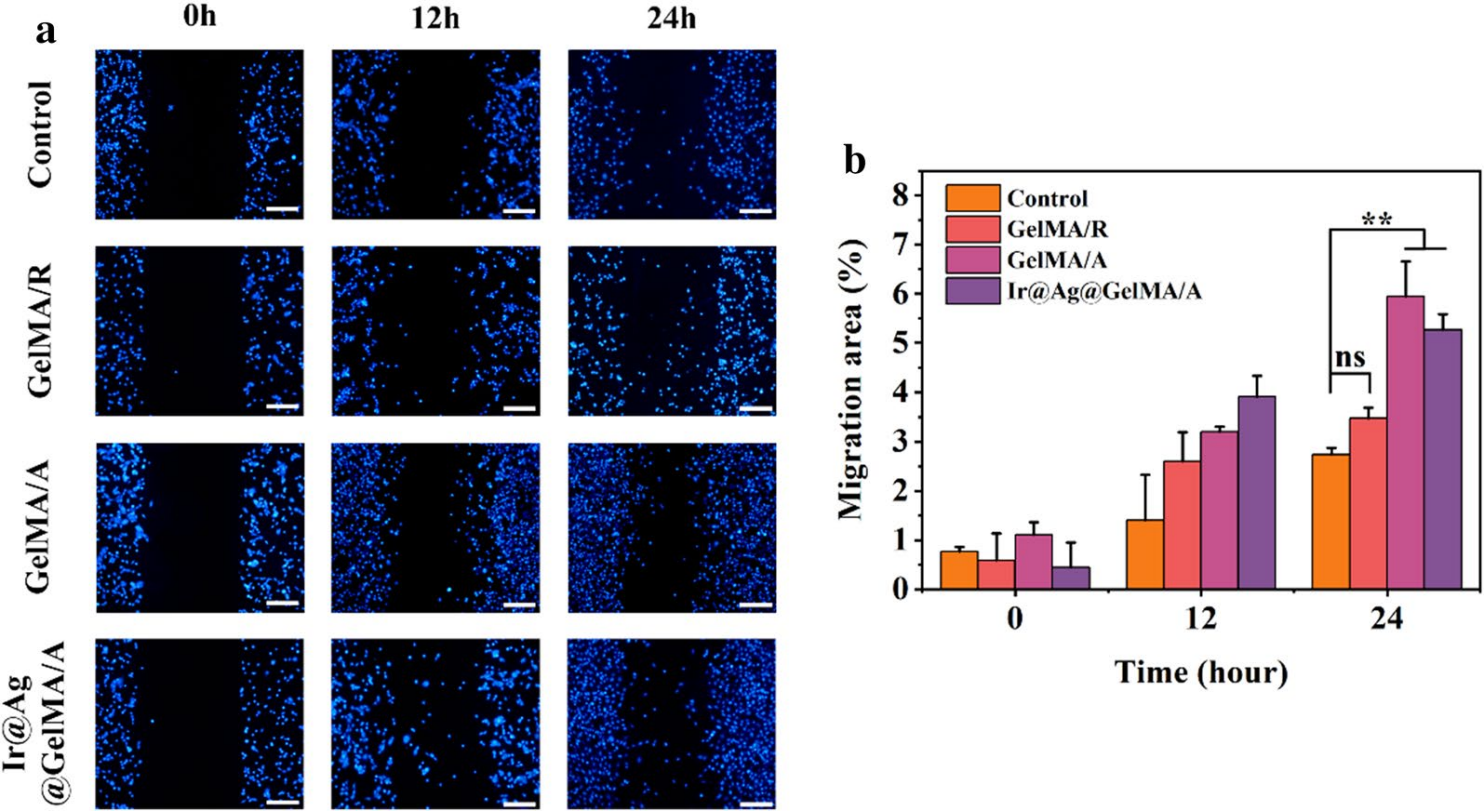
**a** The migration assay of various group. **b** Quantification analysis of migration assay. The scale bars are 200 μm in **a**

Cell migration plays a pivotal role in many complex physiological and pathological processes, especially during wound healing. In order to demonstrate the ability of the composite fiber film for promoting cell migration and its potential influence on wound healing, we applied the non-injury culture-insert assay to study cell migration in vitro. During the 24 h experiment period, the NIH-3T3 cells showed continued migration in each group, and the aligned fiber structure obviously improved the migration speed. Moreover, loading of nanoparticles did not impede such capacity (Fig. 6).

### Ir@Ag@GelMA/A fiber film promotes the healing process of experimental infected wound models

**Fig. 7:**
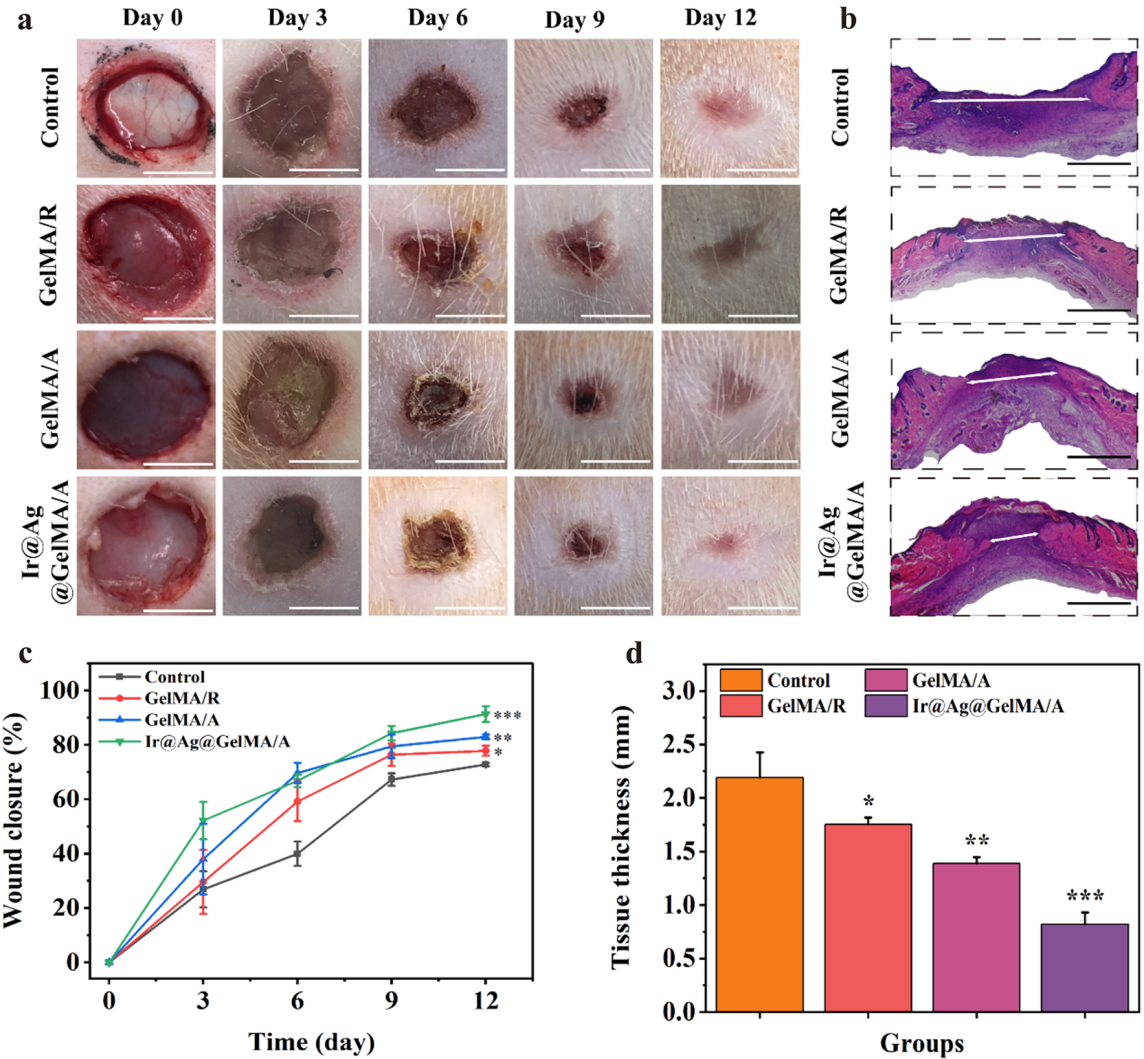
**a** Images of wounds treated with PBS (Control), GelMA/R, GelMA/A, and Ir@Ag@GelMA/A fiber films. **b** Results of H&E staining of Control, GelMA/R, GelMA/A, and Ir@Ag@GelMA/A groups after 12 days. **c** Wound closure rate of various groups (n = 3 for each group). **d** The H&E staining histologic longitudinal sections (n = 3 for each group). The scale bars are 5 mm in **a**, and 1 mm in **b**

To further demonstrate the functional performance of the GelMA fiber film, we applied in vivo experiments using infected wound models (Fig. 7). The rats were modeled on their back skin and added with bacterial suspension. After the infection models being established, all the rats were separated into four groups randomly, treated with PBS solution (Control), GelMA/R, GelMA/A, and Ir@Ag@GelMA/A, respectively. The conditions of each wound were observed and photographed on days 0, 3, 6, 9, and 12 (Fig. 7a). The wound closure rates were also calculated at every point in time compared to the original wound area (Fig. 7c). The results showed that the Control group had the lowest healing rate during the entire experiments, showing a 72.79 ± 0.60% wound closure rate on day 12. The other three groups applied with GelMA fiber films showed a faster healing process than the Control group, with 77.83 ± 1.86%, 82.92 ± 0.95%, and 91.32 ± 2.94% closure rates for GelMA/R, GelMA/A, and Ir@Ag@GelMA/A, respectively. The hydrogel films' excellent mechanical carrier and water retention ability supported cell adhesion and growth, promoting the exchange of nutrients and metabolites, thus accelerating wound healing. Compared with the GelMA/R group, the aligned structure of fibers in the GelMA/A and Ir@Ag@GelMA/A groups was previously verified to guide directional cell growth and improve cell proliferation (Fig. 3a, e), showing a faster closure rate in vivo experiment. Besides, ROS production after wounding will retard the healing process, and infection further exacerbates the wound. The multi-particles doped in the Ir@Ag@GelMA/A group could kill bacteria and eliminate ROS, significantly accelerating wound healing and enhancing wound closure rate. H&E staining of the regeneration skin tissues was applied to further verify the healing results (Fig. 7b). The Ir@Ag@GelMA/A group presented the thinnest granulation tissue of 0.82 ± 0.11 mm, while the Control group possessed the maximum thickness being 2.19 ± 0.23 mm. The thickness in the GelMA/R and GelMA/A groups was also thinner than in the Control group, being 1.75 ± 0.06 mm and 1.39 ± 0.06 mm, respectively (Fig. 7d). These results indicated that encapsulation of PVP-Ir NPs and Ag NPs into GelMA fiber film would improve the wound curing.

### *Ir@Ag@GelMA/A fiber film accelerates the collagen remodeling and neovascularization, inhibits inflammation, and eliminates the *in situ* ROS*

**Fig. 8:**
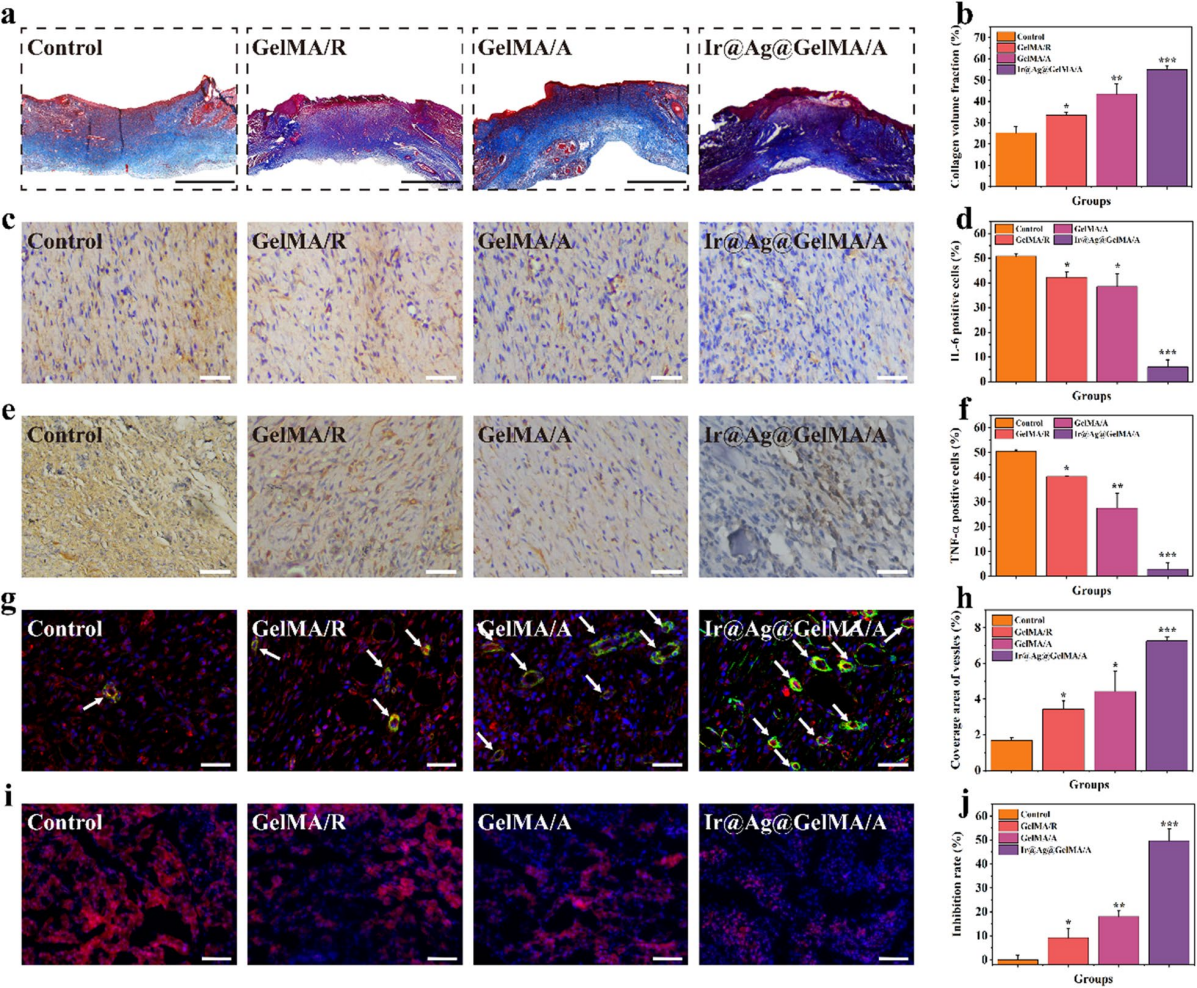
**a** Masson’s trichrome staining in various groups. **b** Quantification analysis of collagen volume fraction. Immunohistochemical results of **c** IL-6 and **e** TNF-α of each group, and the quantification analysis of IL-6 **d**, and TNF-α **f** positive cells, respectively. **g** Double immunofluorescence staining of α-SMA and CD31 for neovascularization. The vessels were pointed with white arrows. **h** Quantification analysis of coverage area of new blood vessels. **i** Double staining images of dihydroethidium (DHE) and DAPI of wounds after different treatments. **j** Quantification analysis of ROS inhibition rate. The scale bars are 1 mm in **a**, and 200 μm in **b–e**

Deposition of collagen at the wound site represents the ultimate stage in the healing process. Therefore, Masson’s trichrome staining measured collagen secretion in all groups (Fig. 8a, b). The cell adhesion and proliferation were supported in the GelMA/R and GelMA/A groups comparing with the Control group, promoting normal fibroblasts’ viability and secretion activity. Because of the scavenging ROS capacity of PVP-Ir NPs and the antibacterial effect of Ag NPs, providing the most suitable physiological environment for fibroblasts, the most abundant deposition of collagen was observed in the Ir@Ag@GelMA/A group. Excessive inflammation caused by skin damage and infection slowed down the closure rate during the curing process. Thus, we detected the expression of the interleukin-6 (IL-6) and tumor necrosis factor-α (TNF-α), using immunohistochemistry experiment on day 12 (Fig. 8c–f). An excess expression of such factors was showed in the Control group, demonstrating a severe inflammation. Conversely, both the GelMA/R and GelMA/A groups expressed fewer inflammatory factors than the Control group, primarily benefiting from the mechanical barrier of the film that prevents further infection. Moreover, a significant reduction of IL-6 and TNF-α was observed in Ir@Ag@GelMA/A because of the synergistic effect of the loaded nanoparticles.

Angiogenesis is another crucial indicator for remolded tissues, and the GelMA fiber films have been demonstrated to promote tissue vascularization. Thus, we tested the expression of CD31 and α-SMA, which were the symbols of the endothelial cell and smooth muscle cell of the vascular, respectively, by double immunofluorescence to further verify the neovascularization (Fig. 8g, h). Results showed that only a few positive staining cells was detected in the Control group, indicating the cells’ impaired migration and differentiation in the severe inflammatory and infection state. In contrast, the GelMA/R and GelMA/A groups showed increased formation of vessels compared to the Control group, and the Ir@Ag@GelMA/A group had the highest number of new blood vessels. The differences may be attributed to the natural properties of GelMA hydrogel fiber scaffold, which has excellent performances in promoting tissue vascularization. To clarify the in situ ROS scavenging activity of nanozymes, the wound site were stained with dihydroethidium (DHE) to measure the expression of ROS (Fig. 8i, j). As shown by fluorescence images, ROS in regenerated tissues was significantly inhibited in the Ir@Ag@GelMA/A group, indicating an excellent antioxidant ability of PVP-Ir NPs loaded fiber film. These results confirmed that the GelMA fiber film with elaborate microstructures and multifunctions promoted wound healing.

## Discussion

The wound scaffold, as a support for cell adhesion and natural physical barrier, is one of the indispensable parts of skin regeneration [10]. The skin’s inherent healing ability is limited, and in most cases, clinical interventions are required to promote wound repair [33]. When a huge wound is formed, the healing process not only needs to ensure the regeneration of skin structure, but more importantly to promote the reconstruction of function, which inevitably requires more complex physiological processes [34–36]. Natural or synthetic skin scaffolds are an effective treatment for curing skin structural and functional disorders. Tissue regeneration engineering has reported that various wound dressings can mediate the physiological regulation mechanism of cytokines and possess improved biocompatibility, accelerating wound healing. In this paper, GelMA hydrogel fiber film was prepared by electrospinning technology. The synthesized fiber film not only had the natural pore structure, which could act as a scaffold for cell adhesion, but also had the characteristics of cellular compatibility and superb water retention ability, simulating the extracellular matrix and facilitating the exchange of the nutrient. In vitro degradation experiments confirmed the self-degradation of hydrogel fiber film, which is one of the prerequisites to ensure its good biosafety. Cell experiments confirmed that electrospun fiber film could promote cell adhesion and proliferation. Animal experiments further confirmed that the synthetic fiber film promoted wound healing.

Tissues such as muscles, blood vessels, and nerves present a uniform cellular arrangement, which plays an important role in maintaining specific functions of organs [37–39]. Therefore, induction of cell orientation is widely considered in tissue regeneration engineering. The scaffolds with the aligned structure not only provide a three-dimensional structural framework, but also controls the morphology, localization, and behavior of cells by mechanically regulating the synthesis of components required for the formation of the cytoskeleton or extracellular matrix, and ultimately promote tissue regeneration [40–42]. By adjusting the rotating speed of the electrospinning receiver, we constructed the aligned fiber film. The results of SEM showed that the parallel arrangement of the nanofibers, and subsequent cell experiments also confirmed that the oriented structure could accelerate cell proliferation and migration compared with the disordered arrangement of the fiber film. Furthermore, animal experiments proved that the aligned fiber film improved wound closure rate.

Reactive oxygen species (ROS), such as hydrogen peroxide, hydroxyl radical, and superoxide anion, generated excessively at the wound site inhibit the activity of physiologically synthesized antioxidant enzymes, leading to severe inflammation and oxidative stress, which is one of the main factors of delayed wound healing [6]. In recent years, nanozymes are a kind of synthetic nanoparticles that can simulate the activity of natural enzymes. As an emerging field, nanozymes have been widely used in biomedical research since their discovery [43]. Persistent infection, which leads to the chronic inflammation in the wound site, is also one of the causes of a refractory wound. At present, antibiotics are mainly used to cure bacterial infections clinically. But due to the long-term overuse, it leads to the emergence of multi-drug resistant bacteria. There are various novel antibacterial agents produced to solve this dilemma, such as metal or metallic oxides nanoparticles, cationic organic and others. Among them, silver have been used as the broad-spectrum antimicrobial agent for a long time, and its derivative, silver nanoparticles, have attracted much attention as both bactericidal and antifungal agent worldwide [44–46]. In this project, iridium nanozymes with the size of 1–3 nm were synthesized by the ethanol reduction method. In combination with the widely used silver nanoparticles with excellent antibacterial effect, a multifunctional electrospinning fiber film doped with nanozymes and silver nanoparticles was prepared. In vitro experiments confirmed that the composite fiber film could scavenge a variety of ROS, protect cells against oxidative injury, and inhibit the growth of bacteria. The nanoparticles doped fiber film showed good biocompatibility and had no obvious inhibition effect on cell proliferation activity. The above results may be due to the self-degradation ability and surface permeability of the hydrogel fiber film. The active substances loaded in the hydrogel fiber film can be absorbed by cells or adhered to the surface of bacteria after contacting the external liquid environment, mediating the corresponding biological activity. In the infected wound models, it was further demonstrated that the multifunctional fiber film inhibited inflammatory response, cleared the in situ ROS, and accelerated the regeneration of blood vessel and granulation tissue, synergistically promoting wound repair. However, although we use the self-degradation properties of the hydrogel fiber film to control the instantaneous release concentration of nanoparticles, ensuring the biological safety to a certain extent, the versatile wound dressing with controlled release ability still need to be designed and proposed, such as photo-responsive hydrogels technology [47], which can accurately control the release timing and concentration of the loading nanoparticles, will truly help nanomedicine translate in clinic.

## Conclusions

In summary, we have proposed an aligned fiber film encapsulated with multi-active ingredients, showing excellent ROS scavenging capability and antibacterial effect for promoting wound healing. Owing to the superb water retention, biocompatibility, and the aligned microstructure of GelMA hydrogel fiber film, the composite dressing was demonstrated to improve cell proliferation, directional extension, and migration. Integrated with the multi-enzymes mimetic PVP-Ir NPs, the composite fiber film could scavenge various ROS. Moreover, the loaded Ag NPs induced a pronounced antibacterial effect of the dressing. And the release concentration of nanoparticles is controlled through the self-degradation property of the hydrogel fiber film, which makes the application of this composite dressing in vivo have better biosafety. We observed the elevated formation of new blood vessels and granulation tissues, benefiting from the properties mentioned earlier. In addition, the inflammation levels at the wound site were reduced due to the functionalized fiber film, accelerating the healing of the wound. These features demonstrated that the synthesized multifunctional film dressing provided a platform for high-performance wound healing materials and showed great potential for the clinically translatable prospect in wound healing.

## Materials and methods

### Materials and animals

IrCl_3_**·**3H_2_O, Polyvinylpyrrolidone (PVP, M_w_ ~ 55,000), Thiazolyl blue tetrazolium bromide (MTT), and Methacrylic anhydride were obtained from Sigma-Aldrich. Hexafluoroisopropanol (HFIP), 3,3′,5,5′-Tetramethylbenzidine dihydrochloride (TMB**)**, and 2-Hydroxy-1-(4′-(2-hydroxyethoxy phenyl)-2-methyl propanone (I2959) were obtained from Aladdin. Catalase (CAT), 2′,7′-dichlorofluorescin diacetate (DCFH-DA) were bought from Beyotime Institute of Biotechnology. 30 wt% Hydrogen peroxide (H_2_O_2_) was obtained from Jinshan Chemical Reagent Factory (China). Silver nanoparticle (15 ± 5 nm) was acquired from XFNANO Tech. Co., Ltd (China). The GelMA hydrogel was synthesized in the laboratory. All male SD rats (200–250 g) were ordered from the Zhejiang Weitong Lihua Laboratory Animal Technology Co., Ltd.

### Synthesis of PVP-Ir NPs

PVP-coated iridium nanoparticles were synthesized by the IrCl_3_·3H_2_O and PVP solution referring to the previous literature. Briefly, 37 mg IrCl_3_·3H_2_O was first added into 50 mL H_2_O and treated with ultrasonic for 2 h until dissolved completely. Next, the above aqueous solution was dropped into 232.5 mg PVP contained 50 mL ethanol solution under stirring. After vigorously stirring overnight, a clear solution was acquired and then refluxed for 8 h at 100 ℃ until a dark brown solution was finally obtained. The excess liquid was vanished by rotatory evaporation and vacuum freeze-drying to obtain ultimate products, finally stored at room temperature for further use.

### Electrospinning of aligned and random GelMA fiber film

GelMA was synthesized in the laboratory by using methacrylic anhydride and gelatin. To prepare aligned GelMA fiber film, 600 mg of freeze-dried GelMA was dissolved with 5 mL HFIP and stirred overnight. The above solution was placed in a syringe with an 18 G tip, then fixed on the micro-pump of the electrospinning machine (YFSPT, Yunfan Technology, China). The voltage was set as 14 kV, the humidity was 30–40%, the distance was set as 10–15 cm between the tip of the syringe and collector, and the pre-gel solution was pumped out at the speed of 4 mL/h. The aligned structure of fiber film was obtained by a high-speed rotating collector with a rotate speed of 2500 rpm/min. Dissolving 0.5 g I2959 into 5 mL alcohol to obtain the photocrosslinking solution. The acquired GelMA fiber film was immersed in the above liquid and exposed to the UV light for 10 min. Then, the fiber film was immersed into alcohol to remove the photoinitiator. The electrospinning process of random GelMA fiber film was similar to the aligned fiber film, except that the rotating speed of the collector was set to 500 rpm/min. To prepare PVP-Ir NPs or Ag NPs loaded GelMA fiber film, various concentrations of the above particles were added into pre-gel and stirring overnight to obtain a homogenous solution. The following process was consistent with the above.

### Characterization

The image of PVP-Ir NPs was captured through the field emission transmission electron microscope (Talos F200S, Thermo Fisher Scientific), and the average size of the nanozymes was obtained by measuring 300 particles based on TEM results by Nano-measurer. The X-ray diffraction spectra (XRD) was measured by the diffractometer (D8 advance, Bruker, German). The FT-IR spectra were obtained by infrared spectroscopy (Tensor II, Bruker, German) between 4000 and 500 cm^−1^. An XPS spectrometer (EscaLab 250Xi, Thermo Fisher Scientific) was used to test X-ray photoelectron spectra (XPS) of the synthesized nanozymes. The UV absorption spectra were obtained by a spectrometer (Cary 3500, Agilent Technologies, USA). The morphology of different GelMA hydrogel fiber film was obtained by scanning electron microscope (SEM, Hitachi SU8010). The PVP-Ir NPs or Ag NPs loaded in fiber film were demonstrated by TEM and EDS analysis. The diameter distributions of different GelMA fibers were obtained by measured 100 random fibers based on the images of SEM using Nano-measure software.

### The CAT-like activity assay of Ir@GelMA/A

The CAT mimic effect of PVP-Ir NPs loaded GelMA hydrogel fiber film was certified by monitoring the change of absorption spectra of H_2_O_2_ at 240 nm for 16 min. Each film was cut into 1 × 1 cm slices, and three replicates were performed for each group. Five different treated groups were set as (I) 20 mM H_2_O_2_, (II) 20 mM H_2_O_2_ + GelMA/A, (III) 20 mM H_2_O_2_ + 1% Ir@GelMA/A, (IV) 20 mM H_2_O_2_ + 2% Ir@GelMA/A, and (V) 20 mM H_2_O_2_ + 3% Ir@GelMA/A. The 5 μg/mL commercial Catalase was set as a positive control to detect CAT activity.

### The POD-like activity assay of Ir@GelMA/A

The POD mimic effect of PVP-Ir NPs loaded GelMA hydrogel fiber film was detected by monitoring the change of absorption spectra of TMB-ox at 652 nm for 8 min. Each film was cut into 1 × 1 cm slices and three replicates were performed for each group. Five different treated groups were set as (I) 5 mM H_2_O_2_ + 1 mM TMB, (II) 1% Ir@GelMA/A + 1 mM TMB, (III) 5 mM H_2_O_2_ + 1 mM TMB + 1% Ir@GelMA/A, (IV) 5 mM H_2_O_2_ + 1 mM TMB + 2% Ir@GelMA/A, and (V) 5 mM H_2_O_2_ + 1 mM TMB + 3% Ir@GelMA/A.

### The SOD-like activity assay of Ir@GelMA/A

The SOD mimic effect of PVP-Ir NPs loaded GelMA hydrogel fiber film was detected by a superoxide dismutase assay kit (S0101S, Beyotime Institute of Biotechnology). Each film was cut into 1 × 1 cm slices, and three replicates were performed for each group. Tests were performed referring to the protocol provided by the commercial manual. Various mass ratios of PVP-Ir NPs loaded films were immersed into the working solution and incubated at 37 °C for 30 min, then the optical density at 450 nm were calculated.

### The hydroxyl radical (·OH) scavenging activity of Ir@GelMA/A

The ·OH degrading ability of Ir@GelMA/A was tested by a Hydroxyl Free Radical assay kit (A018, Nanjing Jiancheng Bioengineering Institute). Each film was cut into 1 × 1 cm slices, and three replicates were performed for each group. Tests were performed referring to the protocol of the commercial manual. Various mass ratios of PVP-Ir NPs loaded films were mixed with the working solution and incubated for 1 min, then the absorbance values at 550 nm were recorded.

### Biocompatible of fiber film

The prepared GelMA/R, GelMA/A, 1% Ir@GelMA/A, 2% Ir@GelMA/A, and 3% Ir@GelMA/A fiber films were divided into discs with an average diameter of 0.5 cm. Then the films were sterilized by being immersed into 75% alcohol with UV-irradiation. After that, the discs were put into 96-well plates, with NIH-3T3 cells seeded on them. Incubated at 37 °C for 1, 3, and 5 days, the optical density (OD_490nm_) was test by standard MTT protocol. And the percentages of the viability were analyzed using following formula: Viability (%) = (OD _samples_/OD _control_) * 100%.

### Cell protection of fiber film against ROS

The prepared GelMA/A, 1% Ir@GelMA/A, and 2% Ir@GelMA/A fiber films were divided into discs with an average diameter of 0.5 cm. The cells were spread on the sterilized films at the concentration of 1 × 10^4^ per well and incubated overnight. Then, 100 μM H_2_O_2_ was dropped to each group and kept for another 24 h. Finally, the cell viability was calculated according to the OD values of each group. To detect the level of intracellular ROS, cells were planted on the sterilized films and incubated overnight. Then, the cells were stained by DCFH-DA in the dark for 30 min. After that, 1 mM H_2_O_2_ was added and incubated for another 15 min. Finally, the cellular ROS levels were determined by fluorescence microscope and flow cytometry.

### Cell alignment on fiber film

The prepared GelMA/R, GelMA/A, and 2% Ir@GelMA/A fiber films were cut into 2 × 2 cm and sterilized by UV-irradiation. The cells were planted on the various films overnight, with the plastic plate setting as Control. The cells were then stained with Calcein-AM for 30 min. The cells on the films were imaged by a fluorescence microscope after being washed with PBS.

### In vitro* antibacterial effect*

The Gram-positive *S.aureus* were incubated to a turbidity of 0.5 based on McFarland standards, then the above bacteria were suspended in sterilized PBS at 1 × 10^7^/mL. Various concentrations of Ag NPs (0–200 μg/mL) and 2% PVP-Ir NPs loaded GelMA fiber films with a diameter of 0.5 cm were put on the bottom of a 96-well plate, adding with 200 μL bacteria suspension. Then measured the optical density (OD) values of each group and recorded as OD_0h_. After being cultured for 24 h, measured again the OD values and recorded as OD_24h_. Furthermore, the suspension of each group was diluted 5 times, then 75 μL of it was spread on the nutrient agar medium plates. The antibacterial activity was verified by colony count after incubation overnight at 37 °C for qualitative analysis, and the changes of OD values for quantitative analysis.

### Migration assay

Cellular migration was detected using a non-injury Culture-Insert 2 Well Assay (ibidi Headquarters Germany). First, the NIH-3T3 cell suspension with a concentration of 3 × 10^5^ cell/mL was obtained. Second, the Culture-Insert 2 Well was put on the various fiber films, and applied 70 μL cell suspension into each well of the Culture-Insert 2 Well, incubating for 24 h until a 100% optically confluent cell layer was formed. Next, removed the Culture-Insert 2 Well gently and added medium to each group, and then started the observation process by taking photos using fluorescence microscope at 0, 12, and 24 h after being stained with DAPI. The number of the cell migrated was calculated and analyzed using the National Institute of Health’s ImageJ software.

### Physical characteristics of fiber film

To test water retention ability, the weight of dry fiber film was calculated as W_0_. After immersing in water for 24 h, the fiber film was placed at room temperature in an open environment, and the weight of each film at 6, 12, 18, 24, 36, and 48 h was calculated as W_t_. The water retention ability (W_R_) was determined by the following formula: W_R_ (%) = (W_t_-W_0_)/W_0_ * 100%. For degradation rate, the initial weight of fiber film was recorded firstly. Then, the film was put into PBS with 100 rpm/min shaking at room temperature. For every 3 days, the weight of fiber film was recorded after freeze-drying, and the ratio to initial weight was calculated as degradation rate. To obtain the mechanical properties of fibers film, each film was cut into 2 × 1 cm flake samples and soaked in PBS for 2 h. Then, the samples were fixed to a universal electronic material testing machine (5944, Instron). The stress-stain curves were obtained by longitudinal stretching at 10 mm/min velocity until failure.

### *Multifunctional hydrogel fiber film for wound healing *in vivo

The infected wound model was established following previous literature. Briefly, the skin on the back with a diameter of 1 cm was removed from each rat after anesthetization. Then, 100 μL bacteria suspension with a concentration of 10^8^/mL was added to the wound area to form an infected wound. After that, the rats were separated randomly into four groups and conducted with PBS (Control), GelMA/R, GelMA/A, and Ir@Ag@GelMA/A fiber films, respectively. The wounds were observed and recorded at day 0, 3, 6, 9, and 12. The rats were sacrificed and anatomized at the terminal of the experiments, and the regenerated skin tissues were collected.

### Histological analysis

The tissues were fixated in 10% paraformaldehyde for 48 h before dehydration. After that, each tissue was dehydrated, embedded, and finally incised into 4 μm thick slices. The obtained tissue pieces were used for Hematoxylin and Eosin staining, Masson’s trichrome staining, and immunohistochemistry experiments. Furthermore, the double immunofluorescence staining of CD31, α-SMA was applied. And the double staining of DHE and DAPI was also operated.

### Statistical analysis

The one-way ANOVA was used to compare differences between multiple groups by the GraphPad Prism 8 software. All the data were presented with mean ± standard deviation. The significances were labeled with *p < 0.05, **p < 0.001, and ***p < 0.0001.

## Supplementary Information

**Additional file 1: Figure S1. **Diameter distribution analysis from 100 random nanofibers of **(a)** GelMA/R, **(b)** GelMA/A, and **(c) **Ir@GelMA/A. **Figure S2. **The ICP-MS analysis of various concentrations of PVP-Ir NPs loaded hydrogel fiber films. **Figure S3. (a) **The UV absorbance spectra of 20 mM H_2_O_2_ solution treated with 5 μg/mL Catalase. **(b) **The UV-vis absorption value at 240 nm of 20 mM H_2_O_2_ solutions treated with 5 μg/mL Catalase. **Figure S4. **The digital pictures of various groups before **(a)** and after **(b)** 5 mM H_2_O_2_ treated. I: Blank + 5mM H_2_O_2_; II: GelMA/A + 5mM H_2_O_2_; III: Ir@GelMA/A + 5mM H_2_O_2_. The red arrows refer to the GelMA/A fiber films, and the blue arrows refer to the Ir@GelMA/A fiber films. **Figure S5.** The concentration of the generated oxygen in Control, GelMA/A, and various Ir@GelMA/A groups. **Figure S6.** The angle distribution of cells growing on the **(a)** Culture plate (Control), **(b)** GelMA/R, **(c)** GelMA/A, and **(d)** Ir@GelMA/A fiber film. **Figure S7.** SEM image of Ir@Ag@GelMA/A fiber film. The scale bar is 2 μm. **Figure S8.** The effect of inducing cell orientation growth of the fiber film before and after loading with Ag NPs.

## Data Availability

The datasets used and/or analysed during the current study are available from the corresponding author on reasonable request.
