# Supplementary material for: Aligned electrospun fiber film loaded with multi-enzyme mimetic iridium nanozymes for wound healing
Source: J Nanobiotechnology. 2022 Nov 16;20:478. doi: 10.1186/s12951-022-01685-2 (PMC9670621; doi:10.1186/s12951-022-01685-2)
Supplement: Supplementary file 1 — Additional file 1: Figure S1. Diameter distribution analysis from 100 random nanofibers of (a) GelMA/R, (b) GelMA/A, and (c) Ir@GelMA/A. Figure S2. The ICP-MS analysis of various concentrations of PVP-Ir NPs loaded hydrogel fiber films. Figure S3. (a) The UV absorbance spectra of 20 mM H2O2 solution treated with 5 μg/mL Catalase. (b) The UV-vis absorption value at 240 nm of 20 mM H2O2 solutions treated with 5 μg/mL Catalase. Figure S4. The digital pictures of various groups before (a) and after (b) 5 mM H2O2 treated. I: Blank + 5mM H2O2; II: GelMA/A + 5mM H2O2; III: Ir@GelMA/A + 5mM H2O2. The red arrows refer to the GelMA/A fiber films, and the blue arrows refer to the Ir@GelMA/A fiber films. Figure S5. The concentration of the generated oxygen in Control, GelMA/A, and various Ir@GelMA/A groups. Figure S6. The angle distribution of cells growing on the (a) Culture plate (Control), (b) GelMA/R, (c) GelMA/A, and (d) Ir@GelMA/A fiber film. Figure S7. SEM image of Ir@Ag@GelMA/A fiber film. The scale bar is 2 μm. Figure S8. The effect of inducing cell orientation growth of the fiber film before and after loading with Ag NPs. [file 12951_2022_1685_MOESM1_ESM.docx]

**Supplementary**

**Aligned Electrospun Fiber Film Loaded with Multi-Enzyme Mimetic Iridium Nanozymes for Wound Healing**

*Boda Wu^1^, Jintao Yang^1^, Yan Zu^2,3,^*, Junjie Chi^3,4^ *, Keqing Shi^1,4,5^ **

1. Translational Medicine Laboratory, The Center of Wound Healing and Regeneration, The First Affiliated Hospital of Wenzhou Medical University, Wenzhou 325035, China
2. Oujiang Laboratory (Zhejiang Lab for Regenerative Medicine, Vision and Brain Health), Wenzhou, Zhejiang 325001, China
3. Engineering Research Center of Clinical Functional Materials and Diagnosis & Treatment Devices of Zhejiang Province, Wenzhou Institute, University of Chinese Academy of Sciences, Wenzhou, Zhejiang 325024, China
4. Cixi Biomedical Research Institute, Wenzhou Medical University, Wenzhou, Zhejiang 325035, China.
5. Key Laboratory of Intelligent Critical Care and Life Support Research of Zhejiang Province, The First Affiliated Hospital of Wenzhou Medical University, Wenzhou 325035, China

* Email: zuyan@foxmail.com

* Email: cjj_2337@163.com

* Email: skochilly@wmu.edu.cn


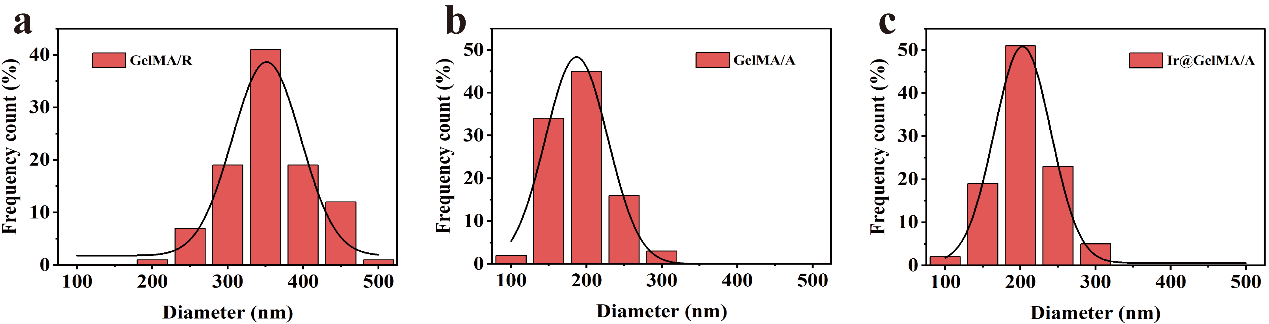
**Figure S1.** Diameter distribution analysis from 100 random nanofibers of **(a)** GelMA/R, **(b)** GelMA/A, and **(c)** Ir@GelMA/A.


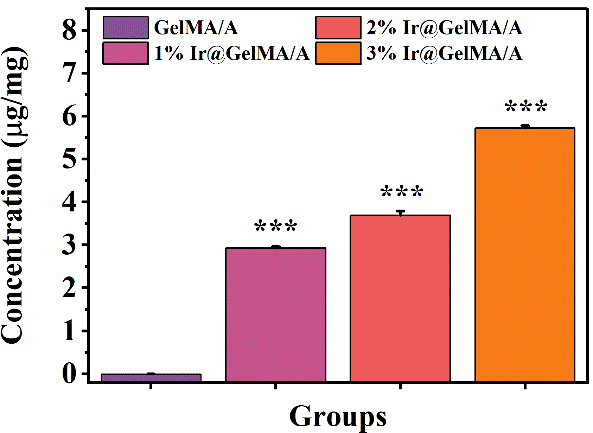


**Figure S2.** The ICP-MS analysis of various concentrations of PVP-Ir NPs loaded hydrogel fiber films.

**
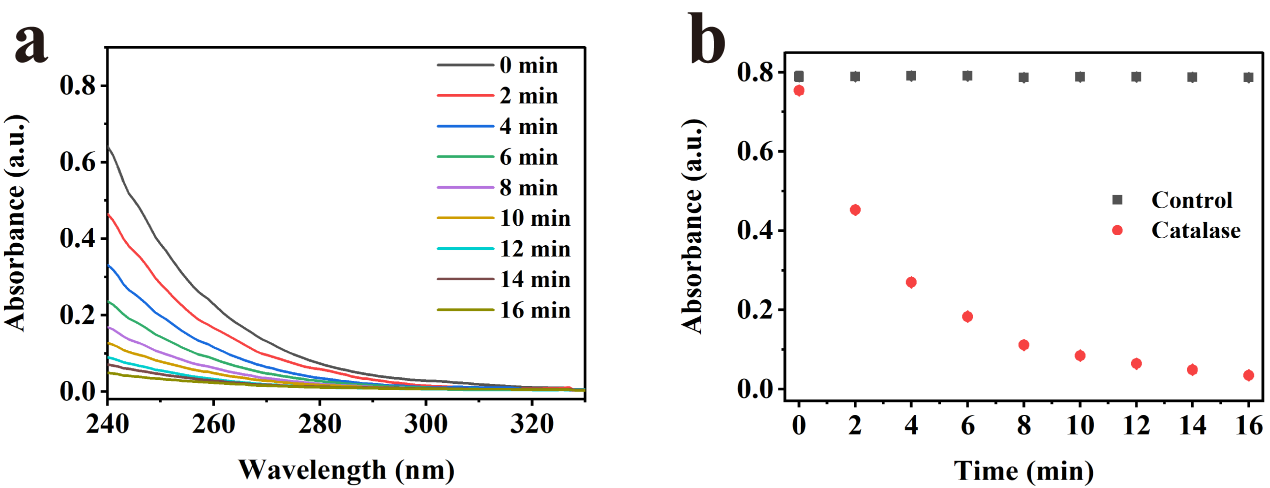
Figure S3. (a)** The UV absorbance spectra of 20 mM H_2_O_2_ solution treated with 5 μg/mL Catalase. **(b)** The UV-vis absorption value at 240 nm of 20 mM H_2_O_2_ solutions treated with 5 μg/mL Catalase.


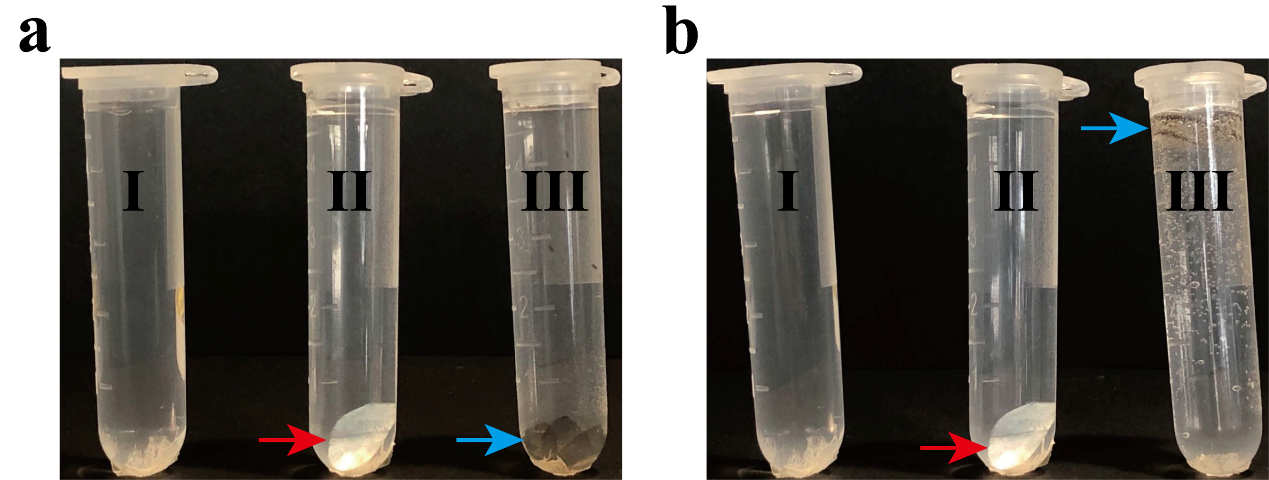
**Figure S4.** The digital pictures of various groups before **(a)** and after **(b)** 5 mM H_2_O_2_ treated. I: Blank + 5mM H_2_O_2_; II: GelMA/A + 5mM H_2_O_2_; III: Ir@GelMA/A + 5mM H_2_O_2_. The red arrows refer to the GelMA/A fiber films, and the blue arrows refer to the Ir@GelMA/A fiber films.





**Figure S5.** The concentration of the generated oxygen in Control, GelMA/A, and various Ir@GelMA/A groups.


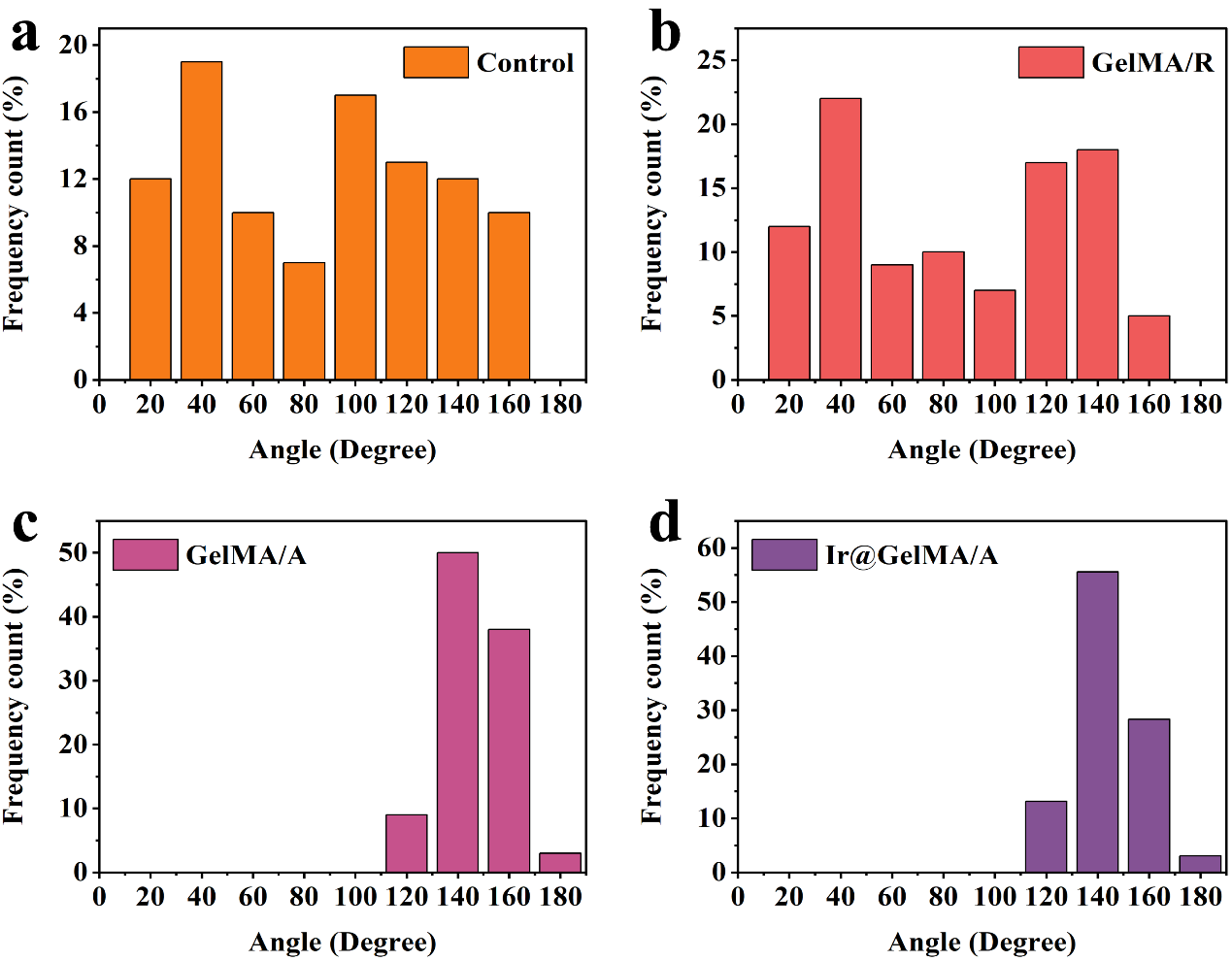


**Figure S6.** The angle distribution of cells growing on the **(a)** Culture plate (Control), **(b)** GelMA/R, **(c)** GelMA/A, and **(d)** Ir@GelMA/A fiber film.


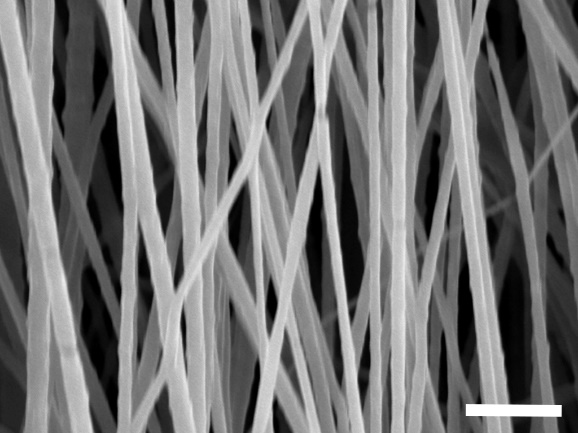


**Figure S7.** SEM image of Ir@Ag@GelMA/A fiber film. The scale bar is 2 μm


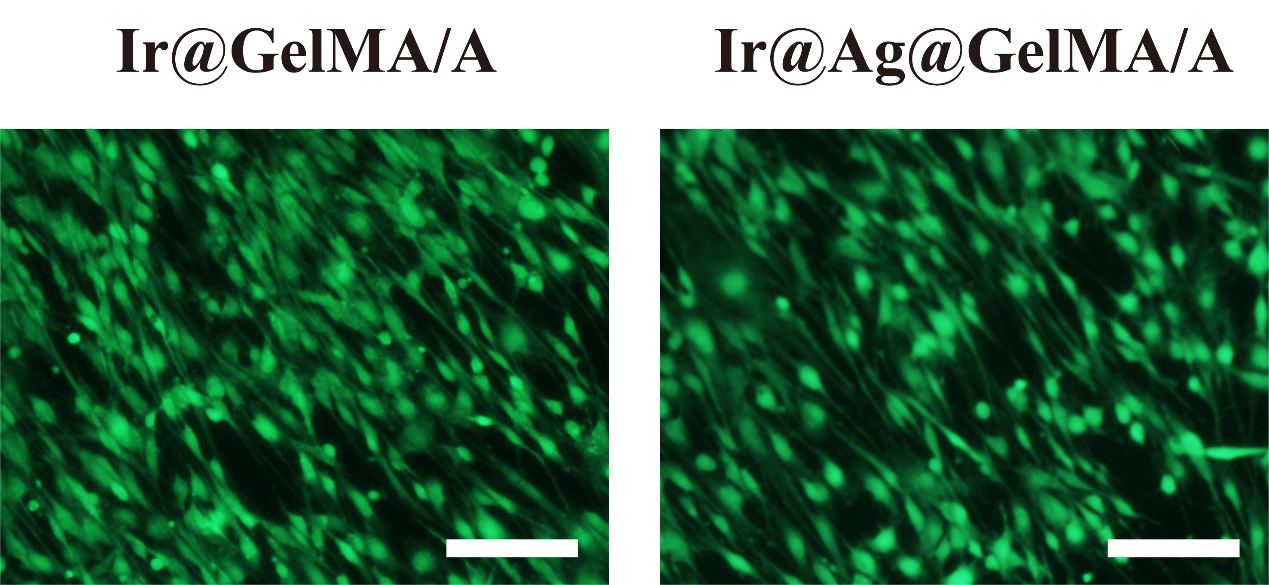


**Figure S8.** The effect of inducing cell orientation growth of the fiber film before and after loading with Ag NPs.
